# The prostate health index PHI predicts oncological outcome and biochemical recurrence after radical prostatectomy - analysis in 437 patients

**DOI:** 10.18632/oncotarget.17476

**Published:** 2017-04-27

**Authors:** Andreas Maxeiner, Ergin Kilic, Julia Matalon, Frank Friedersdorff, Kurt Miller, Klaus Jung, Carsten Stephan, Jonas Busch

**Affiliations:** ^1^ Department of Urology, Charité, Universitaetsmedizin Berlin, Berlin, Germany; ^2^ Department of Pathology, Charité, Universitaetsmedizin Berlin, Berlin, Germany; ^3^ Berlin Institute for Urologic Research, Berlin, Germany

**Keywords:** prostate cancer, biomarker, prostate health index, prostate-specific antigen, biochemical recurrence

## Abstract

The purpose of this study was to investigate the Prostate-Health-Index (PHI) for pathological outcome prediction following radical prostatectomy and also for biochemical recurrence prediction in comparison to established parameters such as Gleason-score, pathological tumor stage, resection status (R0/1) and prostate-specific antigen (PSA).

Out of a cohort of 460 cases with preoperative PHI-measurements (World Health Organization calibration: Beckman Coulter Access-2-Immunoassay) between 2001 and 2014, 437 patients with complete follow up data were included. From these 437 patients, 87 (19.9%) developed a biochemical recurrence. Patient characteristics were compared by using chi-square test. Predictors were analyzed by multivariate adjusted logistic and Cox regression.

The median follow up for a biochemical recurrence was 65 (range 3-161) months. PHI, PSA, [-2]proPSA, PHI- and PSA-density performed as significant variables (p < 0.05) for cancer aggressiveness: Gleason-score <7 or ≥7 (ISUP grade 1 or ≥2) . Concerning pathological tumor stage discrimination and prediction, variables as PHI, PSA, %fPSA, [-2]proPSA, PHI- and PSA-density significantly discriminated between stages <pT3 and ≥pT3 with the highest AUC (0.7) for PHI. In biochemical recurrence prediction PHI, PSA, [-2]proPSA, PHI- and PSA-density were the strongest predictors.

In conclusion, due to heterogeneity of time spans to biochemical recurrence, longer follow up periods are crucial. This study with a median follow up of more than 5 years, confirmed a clinical value for PHI as an independent biomarker essential for biochemical recurrence prediction.

## INTRODUCTION

Prostate-specific antigen (PSA) has been established within the last 30 years for prostate cancer (PCa) management and the detection of its molecular forms including free PSA (fPSA) in the early 1990s [1, 2] enhanced specificity [3]. A fPSA subform named proPSA [4] has proved to be the most cancer-specific PSA form [5, 6]. First data using an in-house assay specific for [-2]proPSA already indicated an association between tumor aggressiveness and this new marker [7, 8].

The introduction of a commercially available [-2]proPSA assay [9, 10] and especially the combined formula [-2]proPSA/fPSA*√PSA named Prostate Health Index (PHI) in 2010 led to clinical improved PCa detection [11, 12]. Numerous multicenter studies in biopsied patients proved that PHI preferentially detects aggressive PCa [13-16]. The FDA approved PHI in 2012 as a biopsy indicator in men with a PSA of 4 to 10 ng/ml and a negative digital rectal examination. A meta-analysis in biopsied men with a PSA of 2 to 10 ng/ml showed a superiority of PHI compared to PSA and percent fPSA (%fPSA) [17].

A prospective study in 350 men who underwent radical prostatectomy (RP) found PHI to be an accurate predictor of pT3 disease, a pathologic Gleason score ≥ 7 (International Society of Urological Pathology (ISUP) grade ≥ 2) and Gleason score upgrading [18]. A recent multicenter study in almost 500 PCa patients with RP indicated a significant accuracy increase in multivariable models by 2.3% and 2.4% for the prediction of pT3 disease and/or pathologic Gleason score ≥ 7 (ISUP grade ≥ 2) by inclusion of PHI, respectively [19]. Consequently, PHI as a confirmed diagnostic tool could be used as a prediction and also as a prognostic tool. To predict the prognosis accurately with focus on cancer control a first indicator is a biochemical recurrence (BCR) defined as PSA increase from zero to 0.2 ng/ml. Further indicators such as metastatic progression or cancer-specific and overall mortality may appear much later than a BCR or may be more important in primary metastatic PCa. However, there is no doubt about the need for a better outcome prediction according to expert opinions [20]. Recommendations regarding adjuvant radiation therapy and/or hormonal treatment after RP, are mainly based on pathological stage (pT2 or pT3), resection status (R0 or R1) and lymph node invasion (pN0 or pN1) [21, 22]. Additional tools including biomarkers [23] for a better evaluation of further therapies could lead to a more individual treatment strategy. This could lead to lower rates of postoperative overtreatment or stronger indications for direct adjuvant postoperative treatment despite an optimal PSA nadir of < 0.01 ng/ml.

While our survey was finished, a very recent study already investigated PHI as a prognostic marker for early BCR [24]. This single center study in 313 RP patients had a short median follow up of 28 months and included only 34 patients with BCR (10.9%). Using an atypical high PHI cutoff of 82, the BCR-free survival rates were 97.7% in those patients below and only 69.7% above this PHI cutoff. However, PHI was an independent predictor of a BCR. In univariate analysis the categorical PHI (cutoff 82) with an area under (AUC) the Receiver Operating Characteristic (ROC) curve of 66.4% was somewhat more accurate than stage (pT3 *vs*. pT2, AUC 66.3%), tumor volume (AUC 65.7%), pathological Gleason score ≥ 7 *vs*. ≤ 6 (ISUP grade ≥ 2 *vs* 1), (AUC 65.5%), surgical margin (resection) status (R1 *vs*. R0, AUC 64.6%) or PSA (AUC 60.1%) [24]. Similarly, a multivariable model that included the categorically coded PHI showed the highest AUC with 71.2% compared to the models based on continuously coded PHI levels (AUC 67.9%) or PSA (AUC 67.3% *P* < 0.001) [24]. However, Lughezzani et al. [24] concluded that external validation in larger populations with longer follow up is needed. Our present study provides information in compliance with this request. The aim of our study was 1) to investigate the predictive power of PHI in comparison with other biomarkers and 2) to compare this serum marker with the established parameters such as Gleason score, stage, margin status or PSA based on longer follow up data for BCR prediction in preoperative and postoperative settings.

## RESULTS

### ROC analysis of PHI

Patient characteristics are displayed within Table 1. During a median follow up of 65 months (range 3-161 months) a BCR was observed in 87 (19.9%) out of 437 included patients. The rate of positive surgical margins (R1) was 26.3% (115) overall, including 40 from 87 cases that developed a BCR. Preoperative risk stratification was performed after d’Amico with 161 (36.8%) low-risk, 219 (50.1%) intermediate-risk and 57 (13.1 %) high-risk cases, respectively. Preoperative median PSA was 4.71 ng/ml (range: 0.25-66.5) and according to d’Amico: < 10ng/ml in 390 (89.2%) cases, > 10ng/ml in 36 (8.3%) cases and > 20ng/ml in 11 (2.5%) cases.

**Table 1:** Patient characteristics

Variable	Included patients *N*=437	BCR *N*=87	No BCR *N*=350	*P*-value * )**)
Age, years Median (Range)	63 (44-78)	64.0 (44.0-75.0)	63 (45.0-78.0)	0.211
PSA, ng/mL Median (Range)	4.71 (0.25-66.5)	5.14 (1.46-66.5)	4.49 (0.00-33.8)	0.092
fPSA ng/ml Median (Range)	0.58 (0.02-10.6)	0.62 (0.00-3.70)	0,58 (0.00-10.6)	0.313
%fPSA/ Ratio Median (Range)	14.1 (3.96-198)	11.7 (3.97-108)	14,5 (4.35-198)	0.064
[-2]proPSA pg/ml Median (Range)	12.1 (0.64-108)	14.4 (2.80-66.9)	11.8 (0.64-108)	0.043
%[-2]proPSA Median (Range)	261 (53.7-1104)	237 (75.2-956)	266 (53.7-1105)	0.312
PHI continous Median (Range)	46.4 (7.79-450)	58.6 (8.17-246)	44.5 (7.79-450)	<0.001
TRUS, mL Median (Range)	35 (12-120)	33.0 (13-114)	35.0 (12.0-120)	0.381
PSA density Median (Range)	0.13 (0.01-1.52)	0.16 (0.36-1.33)	0.12 (0.01-1.52)	<0.001
PHI density Median (Range)	1.33 (0.16-9.38)	1.61 (0.16-9.03)	1.29 (0.24-9.38)	<0.001

Abbreviations: BCR – biochemical recurrence; CI - confidence interval; PSA – prostate-specific antigen; TRUS – transrectal ultrasound; PHI – prostate health index; (*The p-value refers to Fisher’s exact (Chi-square) test for categorical variables; **The p-value refers to Mann Whitney U test for continuous variables).

**Figure 1:**
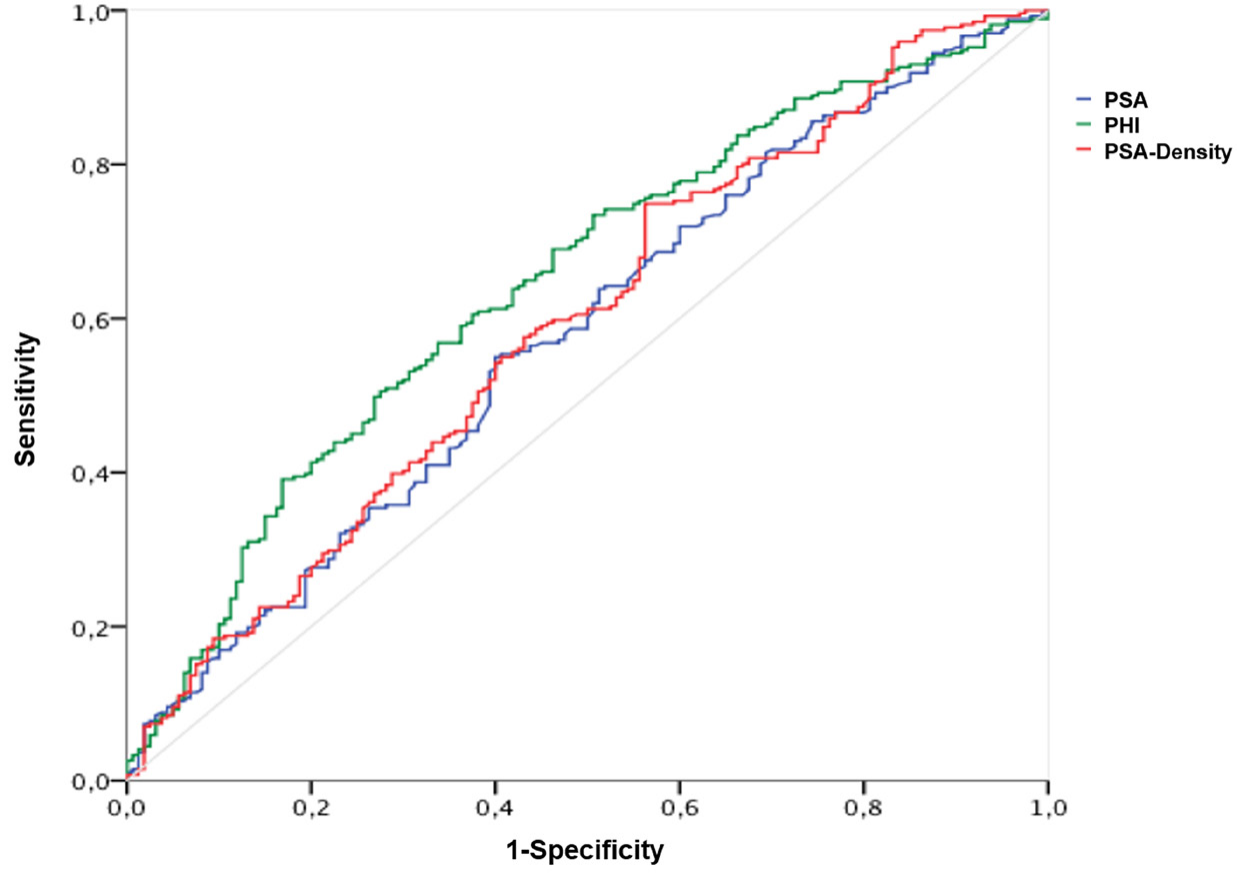
Prediction of postoperative Gleason Score ( < 7 and ≥ 7) Abbreviations: PSA - prostate-specific antigen; fPSA - free PSA; PHI - Prostate Health Index.

**Figure 2:**
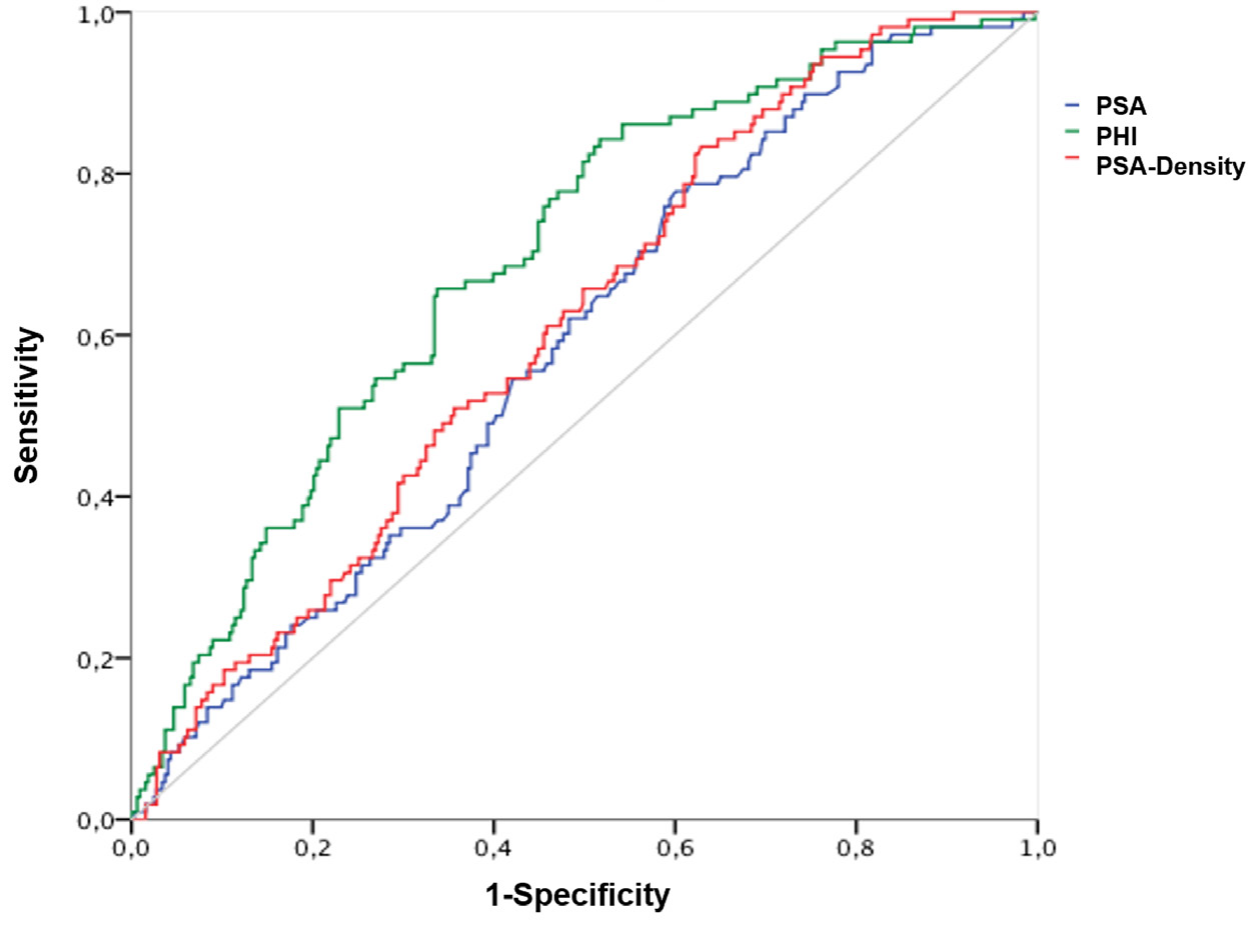
Prediction of pathological tumor stages ( < pT3 and ≥ pT3) Abbreviations: PSA - Prostate specific antigen; fPSA - free PSA; PHI - Prostate Health Index.

**Figure 3:**
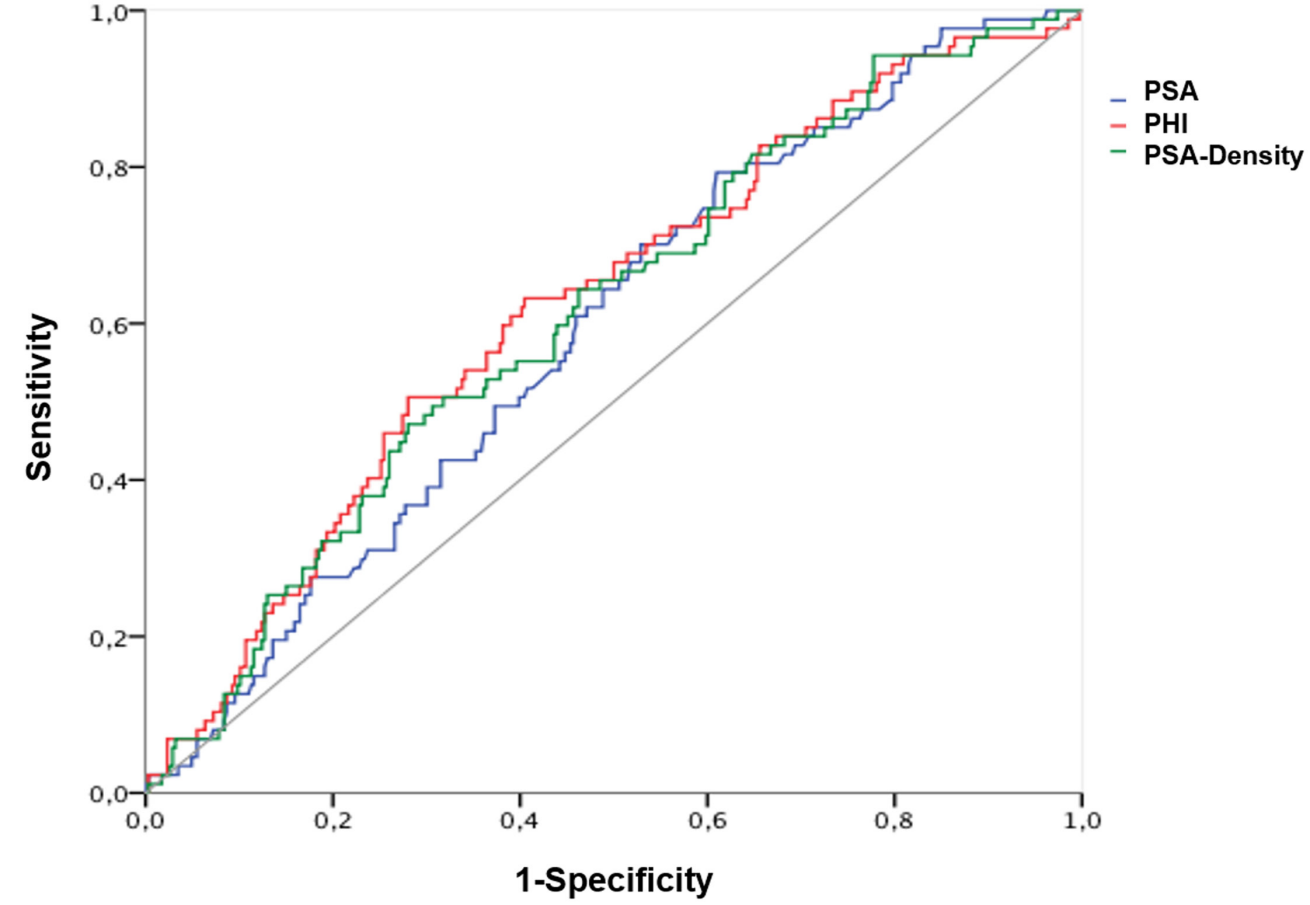
Prediction of biochemical recurrence after radical prostatectomy Abbreviations: PSA - Prostate-specific antigen; fPSA - free PSA; PHI - Prostate Health Index.

The AUC calculations for differentiation between Gleason score < 7 and ≥ 7 (ISUP grade 1 and ≥ 2) are shown in Table 2 with the corresponding ROC curves displayed within Figure 1, respectively. A Gleason score > 7 (ISUP grade ≥ 4) was found in 161 (36.8%); = 7 (ISUP grade 2 and 3) in 220 (50.1%) and < 7 (ISUP grade 1) in 56 (12.8%) patients. Hence, according to aggressiveness (Gleason score < 7 and ≥ 7) (ISUP grade 1 and ≥ 2) PHI, PSA and further [-2]proPSA, PHI- and PSA-density could be identified as significant variables. Contrarily, fPSA with an AUC of 0.528 and %fPSA with an AUC of 0.495 showed no improvement of prediction in comparison to PSA (AUC 0.577). Similarly, [-2]proPSA (AUC 0.597) and % [-2]proPSA (AUC 0.50) showed no additional improvement in contrast to PHI (AUC 0.647). Therefore, fPSA, %fPSA, [-2]proPSA and % [-2]proPSA and PHI-density (AUC 0.616) are not displayed within Figure 1 in order to allow a better overview of relevant variables. The power of differentiation between pT stages ( < pT3 and ≥ pT3) ( < pT3 in 326 (74.6%) and ≥ pT3 in 111 (25.4%) patients) based on the calculated AUC is presented through Table 2 and Figure 2. The variables PHI, PSA, %fPSA and [-2]proPSA and further PHI- and PSA-density were able to differentiate significantly (*p* < 0.05) between < pT3 and ≥ pT3 stage. PHI always performed as the most powerful parameter with an AUC of almost 0.7. Consequently, ROC curves of insignificant parameters have been excluded for a better figure overview. For BCR prediction and differentiation between patients with and without PSA relapse, the ROC-analysis with AUC was also listed in Table 2 and Figure 3, respectively. Concerning the earliest event of BCR, PHI, PSA, [-2]proPSA and similarly PHI- and PSA-density could perform as reliable predictors. Thus, PHI appears consistently in all tested models (Gleason score, pT stage) and currently also in BCR prediction as the strongest parameter. The PHI median of 46.4 provided 10 year BCR-free survival rate of 85.5% below the median and a comparable BCR-free survival rate of 67.7% above our cutoff.

**Table 2:** Predicitive power analysis

	Results	PSA	fPSA	PHI	[-2] proPSA	PSA-density	PHI-density	%fPSA	%[-2] proPSA
Gleason Score <7 / ≥7 (ISUP grade 1 / ≥2)	AUC (95% CI)	0.577 (0.521-0.633)	0.528 (0.472-0.584)	0.647 (0.593-0.700)	0.597 (0.543-0.651)	0.592 (0.536-0.647)	0.616 (0.561-0.670)	0.495 (0.440-0.551)	0.503 (0.447-0.559)
	p-value	0.007	0,333	< 0,001	0.001	0.001	< 0,001	0.867	0.916
<pT3 and ≥pT3	AUC (95% CI)	0.586 (0.527-0.644)	0.518 (0.453-0.583)	0.695 (0.640-0.749)	0.612 (0.549-0.675)	0.611 (0.554-0.668)	0.670 (0.613-0.727)	0.578 (0.358-0.486)	0.527 (0.466-0.589)
	*p*-value	0.007	0.574	< 0,001	< 0,001	0.001	< 0,001	0.015	0.399
BCR YES / NO	AUC (95% CI)	0.591 (0.528-0.653)	0.536 (0.470-0.603)	0.623 (0.559-0.688)	0.573 (0.504-0.641)	0.612 (0.548-0.676)	0.607 (0.540-0.673)	0.564 (0.367-0.505)	0.037 (0.395-0.540)
	*p*-value	0.009	0.293	< 0,001	0.036	0.001	0.002	0.064	0.345

Abbreviations: AUC – area under the curve; PSA – prostate specific antigen; fPSA – free PSA; PHI – prostate health index; pT – pathological tumor stage; BCR – biochemical recurrence; CI – confidence interval;

### Comparison of PHI to established pre- and postoperative outcome parameters

The univariate and multivariate logistic regression model of available preoperative variables as PSA, PHI (continuously coded), PHI median, TRUS volume, age and biopsy Gleason score predicting pathological stage > pT2 were summarized in Table 3. Even though the performance of PHI using a cutoff value of 46.4 was inferior to the biopsy Gleason score, an odds ratio of 2.86 (*p* < 0.00001) for the univariate and 2.20 (*p* = 0.001) for the multivariate approach was significantly higher in comparison to all other parameters.

**Table 3:** Univariate and multivariable logistic regression model of preoperative variables predicting pathological stage >pT2 following radical prostatectomy

Variable	Univariate analyses			Multivariable analyses		
	OR	95% CI	*p*-value	OR	95% CI	*p*-value
PSA, ng/mL (continuous)	1.04	0.99-1.09	0.070	n/a		
PHI (continuous)	1.02	1.01-1.03	<0.00001	n/a		
PHI >median vs <median	2.86	1.80-4.53	<0.00001	2.20	1.36-3.57	0.001
TRUS volume, ml	0.99	0.98-1.00	0.121	n/a		
Age, years (continuous)	1.02	0.99-1.06	0.18	n/a		
Biopsy Gleason Score <7 (ISUP grade 1) =7 (ISUP grade 2 and 3) >7 (ISUP grade ≥4)	Ref 4.11 10.86	2.24-7.53 5.13-22.99	<0.0001 <0.0001	Ref. 3.65 8.76	1.97-6.73 4.08-18.82	<0.00001 <0.00001

Abbreviations: PSA – prostate-specific antigen; PHI – prostate health index; pT – pathological tumor stage; TRUS – transrectal ultrasound; CI – confidence interval; OR – odds ratio; CI - confidence interval.

The results of logistic regression models that tested the prediction of a Gleason score > 6 (ISUP grade ≥ 2) are shown in Table 4. The performance of PHI was superior in comparison to all other parameters including clinical stages with consecutively higher odds ratios of 2.46 and 2.09.

**Table 4:** Univariate and multivariable logistic regression model of preoperative variables predicting pathological Gleason Score >6 following radical prostatectomy

Variable	Univariate analyses			Multivariable analyses		
	OR	95% CI	*p*-value	OR	95% CI	*p*-value
PSA, ng/mL (continuous)	1.07	1.01-1.13	0.015	n/a		
PHI (continuous)	1.02	1.01-1.03	0.00002	n/a		
PHI >median vs <median	2.46	1.64-3.67	<0.0001	2.09	1.35-3.24	0.001
Clinical stage ≥cT2 vs cT1	1.95	1.12-3.16	0.006	1.82	1.11-2.96	0.017
Age, yrs (continuous)	1.014	0.99-1.05	0.341	n/a		
TRUS volume, ml	0.997	0.99-1.01	0.570	n/a		

Abbreviations: PSA – prostate-specific antigen; PHI – prostate health index; pT – pathological tumor stage; TRUS – transrectal ultrasound; CI – confidence interval; OR – odds ratio; CI - confidence interval.

Table 5 reflects the findings of the univariate and multivariate Cox-regression of postoperative variables predicting biochemical recurrence. PHI showed substantial results with a hazard ratio of 1.83 within the univariate approach and 1.57 for the multivariate analysis, respectively. Controversially PHI showed an inferior performance to established parameters such as Gleason score, T stage, margin status or PSA.

**Table 5:** Univariate and multivariable Cox-proprotional Hazard model of postoperative variables predicting biochemical recurrence following radical prostatectomy

Variable	Univariate analyses			Multivariable analyses		
	HR	95% CI	*p*-value	HR	95% CI	*p*-value
PSA, ng/mL (continuous)	1.051	1.02-1.08	0.001	n/a		
PHI (continuous)	1.007	1.003-1.011	<0.001	n/a		
PHI >median vs <median	1.83	1.16-2.89	0.009	1.57	0.98-2.49	0.060
Pathological stage ≥pT3a vs <pT3a	2.76	1.76-4.31	<0.0001	1.76	1.11-2.80	0.017
Margin status R1 vs R0	2.55	1.63-3.99	<0.0001	1.50	0.92-2.44	0.101
Age, yrs (continuous)	1.03	0.99-1.07	0.167	n/a		
Pathological Gleason Score <7 (ISUP grade 1) =7 (ISUP grade 2 and 3) >7 (ISUP grade ≥4)	Ref 2.06 5.40	1.15-3.67 2.91-10.13	0.015 <0.0001	Ref 1.92 3.73	1.09-3.40 1.94-7.14	0.025 <0.0001

Abbreviations: PSA – prostate-specific antigen; PHI – prostate health index; pT – pathological tumor stage; TRUS – transrectal ultrasound; CI – confidence interval; HR – hazard ratio; CI - confidence interval.

## DISCUSSION

In our final cohort with available 437 patients (95% of 460 operated men) and complete follow up after RP from 2001 to 2014 we first represent PHI as a prognostic biomarker based on long term data. While most patients develop a BCR within the first two years, some have a recurrence of up to five years after RP [25]. We believe that a sufficient prediction regarding a possible BCR can only be made after a median follow up of at least 5 years because the number of BCR cases in the years 5 to 10 after RP are relatively low and decreasing continuously [25]. The data of this study supports this since 83.2% are recurrence free after 5 years, 78.6% after 8 years and 77.2% are recurrence free after 10 years. Only 6% of all PCa patients develop a BCR within the years 5 to 10 after RP.

However, there is a clear need for better prognostic factors after RP to outweigh further treatment options like radiation or even hormonal treatment because not all patients with adverse pathology do develop a BCR. Here PHI could show its clinical value with a significantly higher median of 58.6 in those 87 patients with BCR as compared with 45.0 in those 350 men without PSA relapse (*p* = 0.011). Lughezzani et al. [24] did not provide these median values seeing as their study only included 34 patients with a BCR. They proposed a very high PHI cutoff of 82 to obtain a BCR-free survival rate of 97.7% in the patients below 82 and 69.7% for those above this cutoff [24]. When using our data, a differentiation with the PHI median of 46.4 provided 10 year BCR-free survival rate of 85.5% below the median and a comparable BCR-free survival rate of 67.7% above our cutoff. The somewhat decreased percentage of patients without BCR (85.5% *vs*. 97.7%) is most likely a result of the clearly longer follow up (65 *vs*. 28 months) in our study. When using the median PHI value, the difference between both BCR-free survival rates is lower (83.5% *vs*. 70%), which indicates that PHI is able to predict a recurrence situation. This is also evident when comparing the AUCs of all biomarkers between patients with and without BCR. Here, PHI had the largest AUC of 0.62 compared with PSA (0.59) or %fPSA (0.56). Furthermore, PHI could also best separate between Gleason < 7 and Gleason ≥ 7 (ISUP grade 1 and ≥ 2) with an AUC of 0.65 compared with PSA (0.58) and percent free PSA (0.51). This confirms the ability of PHI to predict tumor aggressiveness in pathological prostatectomy specimen, as already published before [19].

Accordingly, the performance of PHI within our applied logistic regression models showed its power in both preoperative as well as postoperative settings. As a consequence, patients could benefit from its clinical value before receiving adjuvant treatment or unnecessary invasive procedures preoperatively like for example repeat biopsies as recently published concerning men with a total PSA > 10 ng/ml [26].

In distinction to PHI, several biomarkers for an improved BCR estimation have been reviewed [23] but none of these other markers is used in a clinical routine setting. Interestingly, a biopsy-based 17-gene genomic prostate score recently predicted a BCR and also adverse pathological outcome in men with low- and intermediate-risk PCa [27]. However, the likelihood for PHI as a routine serum parameter to become an additional tool for long term prediction seems to be much higher than for experimental marker. Particularly, PHI outperformed PCA 3 and the inclusion in Epstein and PRIAS protocols could show its substantial contribution to prediction of insignificant cancer and better selection of active surveillance candidates [28].

While Lughezzani et al. [24] used the traditional Hybritech calibration for PSA and fPSA to obtain their PHI values, our PSA and fPSA measurements were all performed using the WHO calibration that provides about 20-25% lower PSA values [29]. The differences in PHI however were almost negligible as seen in a study using both calibrations [30].

The PHI seems to improve the overall prediction and herewith claims its important stance in clinical decision making. Despite a positive role for PHI in PCa prediction, we acknowledge limitations to the present study. Primarily our study represents a single center analysis with a retrospective approach whereas prospective multicenter studies are desirable due to a higher impact. In addition, after a 10 year follow up the categorical data of pT stage ( < pT3: BCR free 83.5% *vs*. ≥ pT3: BCR free rate 60.1%) and Gleason score (Gleason 6 (ISUP grade 1): BCR free 90.1%, Gleason 7 (ISUP grade 2 and 3): BCR free 76% and Gleason ≥ 8 (ISUP grade ≥ 4): BCR free rate 47.4%) showed larger differences than PHI (85.5% *vs*. 67.7%), which indicates that PHI independently should not be used as predictor. We are aware that time dependent functions (especially survival outcomes) can be displayed by Kaplan-Meier curves. While survival was not the primary endpoint but BCR, the AUC represents a reliable estimate. Since lymphadenectomy was only performed in 267 patients, the lymph node status was only applicable in 61.1%. A median of 14 lymph nodes were taken and positive lymph nodes were reported only in 5 (1.87%) patients. Consequently no analysis of the role of lymphadenectomy for BCR within our cohort was performed. A current important review from the EAU prostate cancer panel obtaining 66 studies with a total of 275269 patients analyzed the benefits and harms of the different extents of lymph node dissection. Meaning lymph node removal enables accurate assessment of cancer spread but may not have any direct benefit on cancer outcomes [31].

Calibration plots and decision curve analysis have not been included in our analysis, as once requested by Nguyen and Kattan in order to identify the true clinical value of a given marker as the ultimate goal [32]. But Nguyen and Kattan also requested follow-up studies involving long-term use of a marker in a multivariable analysis [32].

In conclusion, this study confirmed a clinical value for PHI in prediction of a BCR by using a multivariable approach with a median follow up of more than 5 years.

## MATERIALS AND METHODS

### Study design and population

A total of 460 PCa patients with preoperative PHI measurements with WHO calibration (Beckman Coulter Access 2-Immunoassay) undergoing RP (open, laparoscopic or robot-assisted) and pelvic lymph node dissection between 2001 and 2014 at a single German tertiary center were identified. Blood was drawn and serum samples were prepared and frozen at -80°C within 3h of blood collection according to recommendations for pre-analytic tPSA and fPSA and p2PSA as previously published [16]. All pre-operative, operative and follow-up data were collected under an internal review board (Charité ethical committee)-approved protocol and after obtaining written informed consent from all patients. Standardized, self-administrated questionnaires were routinely sent out to all patients after 3 months and subsequently year by year after radical prostatectomy. All men were invited to complete a questionnaire consisting of the International Prostate Symptom Score (IPSS), the International Index of Erectile Function (IIEF-5), the International Consultation on Incontinence Questionnaire (ICIQ), question 29 and 30 of the European Organization for Research and Treatment of Cancer Quality of Life Questionnaire (EORTEC QLQ-30) and general data about date of surgery, level and date of the last estimated prostate-specific antigen.

BCR was defined as two consecutive PSA values > 0.1 after a previous non-detectable level. Consequently, time to BCR was defined as months between surgery and BCR development and concerning patients without BCR, censoring was performed at the time of last follow-up. All patients with preoperative anti-androgen therapy and with persisting PSA levels after RP were excluded from the analysis. Data on recurrences and survival was obtained *via* telephone interviews and standardized questionnaires.

### Statistical analysis

Differences between patients with and without BCR were compared using the chi-square (Fisher’s exact) test for categorical variables and the Mann Whitney U-Test for continuous variables. Gleason score, pathological tumor stage, and surgical margin status, were considered as categorical variables. PHI, Age and PSA were regarded as continuous variables. The power of prediction was analyzed multivariate adjusted logistic and Cox regression. All statistical calculations were two-sided unless stated otherwise and performed using SPSS v.23 (IBM Corp, Somers, NY, USA). A *p*-value < 0.05 was considered statistically significant.

## Abbreviations

PSA: prostate-specific antigen
PCa: prostate cancer
fPSA: free prostate-specific antigen
PHI: prostate health index
%fPSA: percent free prostate-specific antigen
RP: radical prostatectomy
ISUP: international society of urological pathology
BCR: biochemical recurrence
pT: pathological tumor stage
pN: lymph node invasion
AUC: area under the curve
ROC: receiver operating curve
R: resection status
